# REGIONAL VARIATION IN COVID-19 MITIGATION PRACTICES AMONG HOME- AND COMMUNITY-BASED LONG-TERM CARE PROVIDERS

**DOI:** 10.1093/geroni/igac059.1801

**Published:** 2022-12-20

**Authors:** Jessica Lendon, Christine Caffrey, Priyanka Singh, Zhaohui Lu, Sengupta Manisha

**Affiliations:** National Center for Health Statistics, Hyattsville, Maryland, United States; National Center for Health Statistics, Hyattsville, Maryland, United States; National Center for Health Statistics, Hyattsville, Maryland, United States; National Center for Health Statistics, Hyattsville, Maryland, United States; National Center for Health Statistics, Hyattsville, Maryland, United States

## Abstract

Residential care communities (RCC) and adult day services centers (ADSC) were greatly impacted by COVID-19. As state-regulated home- and community-based care settings (HCBSs), they experienced changing regulations, outbreaks among care recipients and staff, and new challenges to safely provide services. Using a nationally representative sample of over 11,600 RCCs and census of nearly 5,500 ADSCs from the 2020 National Post-acute and Long-term Care Study, this study examined the prevalence and regional variation (p<.05) of practices to mitigate COVID-19. Preliminary data show that most RCCs (87%) and ADSCs (72%) screened for symptoms. Most RCCs limited communal activities (83%) and ADSCs reduced hours or temporarily closed (73%). More RCCs used video telemedicine than audio-only (40% vs 34%) to assess, diagnose, or treat users. However, more ADSCs used audio-only than video telemedicine (25% vs 17%). Over 80% of all providers always or sometimes imposed in-person restrictions on family, visitors, volunteers, and non-essential services providers. More ADSCs in the Midwest (82%) and South (80%) screened symptoms than Northeast (68%) and more ADSCs in the Midwest (85%) limited hours/temporarily closed than Northeast (72%). More RCCs in the Midwest (43%) and South (41%) used video telemedicine than Northeast (36%); however, a lower percentage of ADSCs in the Midwest (9%) and South (12%) used video telemedicine than Northeast (25%). Results show a variety of experiences in the first year of the COVID-19 pandemic among these two HCBSs. Practices to mitigate COVID-19 while continuing to provide needed services were common, with some differences across settings and US regions.

